# Health-Related Quality of Life in Italian Adolescents During Covid-19 Outbreak

**DOI:** 10.3389/fped.2021.611136

**Published:** 2021-04-29

**Authors:** Francesca Mastorci, Paolo Piaggi, Cristina Doveri, Gabriele Trivellini, Anselmo Casu, Marta Pozzi, Cristina Vassalle, Alessandro Pingitore

**Affiliations:** ^1^Clinical Physiology Institute, National Research Council, Pisa, Italy; ^2^Department of Information Engineering, University of Pisa, Pisa, Italy; ^3^Department of Addictions, Azienda Sanitaria Friuli Occidentale, Pordenone, Italy; ^4^Fondazione G. Monasterio, Regione Toscana, Pisa, Italy

**Keywords:** quarantine, COVID-19, health, well-being, adolescent, gender differences

## Abstract

Coronavirus disease 2019 (COVID-19) outbreak represented an experience of social isolation potentially leading to changes in the health quality of life. The aim of this study is to investigate the health-related quality of life during quarantine in early adolescents. Data were collected from 1,289 adolescents (mean age, 12.5; male, 622), at the beginning of the school year (September 2019, Standard Condition, SC) as part of the AVATAR project and during Phase 1 of the Italian lockdown (mid-late April 2020) (COVID-19 Quarantine, CQ) using an online questionnaire. In the CQ period, with respect to SC, adolescents showed lower perception in the dimensions, such as psychological (*p* = 0.001), physical well-being (*p* = 0.001), mood/emotion (*p* = 0.008), autonomy (*p* = 0.001), and financial resources (*p* = 0.018). Relationship with the family (*p* = 0.021) and peers (*p* = 0.001), as well as the perception of bullying (*p* = 0.001) were reduced. In lifestyle, adolescents developed higher adherence to the Mediterranean diet (*p* = 0.001). Adolescents living in the village had greater reduction in both autonomy (*p* = 0.002) and peer relationships (*p* = 0.002). Moreover, the perception of physical well-being was lower in those living in the city instead of those living in the countryside (*p* = 0.03), in an apartment instead of a detached house (*p* = 0.002), and in those who did not have green space (*p* = 0.001). Gender effect emerged for the psychological (*p* = 0.007) and physical well-being (*p* = 0.001), mood/emotion (*p* = 0.001), and self-perception (*p* = 0.001). The study showed that health-related quality of life during quarantine changed in its psychosocial dimensions, from mood and self-esteem to social relationships, helping to define the educational policies at multiple points in the promotion process of health.

## Introduction

Coronavirus disease 2019 (COVID-19) outbreak represented a unique experience of social isolation and spatial confinement that is a no-laboratory, but a naturalistic model involving millions of people of different age, culture, and social context, provoking, definitely, relevant, fast, and unforeseen changes in daily lifestyle habits (1). This was particularly true for children and adolescents who had to get used to homeschooling, dealing with the lack of their usual daily environment, made by direct contact with classmates, friends, and teachers. In addition, they have lost their spare time activities, are forced to live in the restricted home environment, and have to share common spaces with the other family members. Actually, the COVID-19 quarantine (CQ), with the forced confinement, isolation, and privation, represents a multidimensional stress factor that can give rise to short- and long-term consequences on physical and mental health. The psychological effects of the quarantine from SARS, Ebola, and H1N1 outbreaks were focused on long-term outcomes in adult population, especially in healthcare professionals, documenting exhaustion, detachment from others, anxiety, irritability, insomnia, poor concentration, and deteriorating work performance (2, 3). In particular, 3 years after the SARS outbreak, the substance abuse and avoidance behaviors found in the healthcare professionals were also positively related to quarantine (1, 4). While psychological effects in adults are well-documented, not much is known about the health effects of outbreaks on adolescents. In fact, adolescence is characterized by significant changes in brain development, emotions, cognition, behavior, and interpersonal relationships (5).

Only one study, in children, evaluated the psychological impact of the quarantine as a predictor for long-lasting post-traumatic stress symptoms, reporting four times higher probability in its development than in those who were not quarantined (6).

Most of the evidence in this field comes from animal models in which social isolation, considered as stress paradigm, resulted in long-term alterations in social behavior, neurochemical and neuroendocrine systems, and variations in neuroplasticity-related genes (7–9). In particular, animal research has shown that social deprivation associated with isolation in adolescents induced exclusive effects on brain development and behavior compared to other phases of life (10).

Adolescence, in general, is considered the healthiest time of life, characterized by many of the requisite components needed for ideal health (11); although epidemiological data showed that despite behavioral risk factors that might not affect health status during this period, they can have crucial effects later in life (12). According to this perspective, a good health status is predominantly linked to four lifestyle habits (smoking status, body mass index, physical activity, and diet). In addition to these factors, epidemiological and neurobiological studies showed that health and well-being in adolescence are linked to other variables belonging to the psychosocial dimension, including positive social relationships (13–15). For this reason, the variables considered here have been previously described as influencing the health status and well-being of adolescents (13–15). Adolescence is also a time of increasing behavioral divergence between males and females (16–19). In general, female adolescents have a poorer perception of their own health, a lower level of life satisfaction in the psychosocial context and an increased frequency of somatic symptoms than male adolescents (19).

The hypothesis of the present study is if quarantine is to be considered only as a period of crisis for adolescents who live in a continuous interchange between resilience and vulnerability, or if it can even become a moment of emotional, cognitive, and social reset, ending up in transforming a critical period to a psychosocial resource.

Therefore, the current study investigated health-related quality of life (physical, psychological, family and peer relationships, autonomy, homeschooling, sleep quality, and lifestyle habits) in early Italian adolescents during Phase 1 of the Italian lockdown. This was possible using a web platform (www.euroavatar.eu) based on a wide range of data integration, already used by high schools joining the AVATAR (A new purpose for the promotion and evaluation of health and well-being among healthy teenagers) project, in order to monitor and promote the perception of adolescents on health and well-being (20–22).

## Methods

### Participants

During the school year 2019/2020, in September/October, 3,458 students were monitored, and 1,289 of these completed the questionnaires during the lockdown. Therefore, the final population consisted of 1,289 early adolescents (mean age, 12.5; male, 622), with data acquired in SCs (at the beginning of the school year) and during COVID-19 quarantine.

Adolescent students were enrolled according to the following inclusion criteria: age 10–14 years, absence of neuropsychiatric or other diseases, informed consent signed, and filling of the entire questionnaires proposed.

### Study Design

The survey, AVATAR COVID-19, was conducted, during Phase 1 of the Italian lockdown (mid-late April 2020), using an online questionnaire. Ten junior high schools (middle schools) participated in the AVATAR COVID-19 study; a section of AVATAR project aimed to develop a new tool to assess the lifestyle habits, social context, emotional status, and mental skills in adolescents, and to define an integrated index of the best indicators of well-being (20–22).

Schools that participated in the survey were located in Central and Northern Italy, mainly in Tuscany (seven schools), one in Liguria, and two in Friuli Venezia Giulia. The choice of schools depended on their voluntary participation in the project. According to the AVATAR approach, data were usually collected at the beginning and at the end of the school year, in order to allow the teachers to evaluate the effectiveness of educational strategies defined on the basis of the identified needs. In every class from every school, all the adolescents filled out the questionnaire. They filled out questionnaires in two different phases: SC, at the beginning of the school year, and CQ, during the lockdown phase.

Participants were previously instructed on how to fill out the questionnaires and how to conduct the tests. During the first monitoring, the tests were conducted during the computer class, during school time, while in quarantine, the tests were completed at home during the distance-learning period, in the presence of a teacher. No incentive was provided to the adolescents or parents. A research assistant was available to provide information and technical support to complete the questionnaires in both the conditions. The study was approved by the internal ethics committee of each participating school, in accordance with Italian law. In addition, all parents or legal guardians gave informed consent, and authorized the researchers to use their data in accordance with the Italian law. All procedures performed in the study were in accordance with the ethical standards of the institutional and/or national research committee and with the 1964 Declaration of Helsinki and its later amendments or comparable to ethical standards.

### Procedures

Data were collected with the AVATAR web tool (20). Before completing the questionnaires, the students participated in a training session on the meaning of the project and the terminology used. A record of the socio-demographic data was used to collect the information on gender, age, schooling, family structure, and body mass index, according to the age group of WHO (23). The Italian version of KIDSCREEN-52 was used to assess the health-related quality of life (24, 25). The KIDSCREEN is a self-report questionnaire designed to address the health-related quality of life. The questionnaire, which describes the physical, psychological, mental, social, and functional aspects of well-being, consists of 52 items grouped into 10 dimensions [physical well-being, psychological well-being, moods and emotions, self-perception, autonomy, parent relations and home life, social support and peers, school environment, social acceptance (bullying), and financial resources]. Some sample items include the following: “*In general, how would you say your health is?”* for the physical well-being dimension; “*Have you felt satisfied with your life?*” for moods and emotions; “*Have you been happy with the way you are?”* for self-perception. Cronbach's alpha ranges from 0.77 to 0.89 for the dimensions of the 52-item version. The KIDSCREEN questionnaires are psychometrically tested using the data obtained from a multicenter European study that included a sample of 22,827 children recruited in 13 countries (26).

Dietary habits were evaluated using the Mediterranean Diet Quality Index for children and adolescents (KIDMED) (Cronbach's alpha = 0.79, 95% CI: 0.71–0.77) (27). The KIDMED index was based on principles sustaining or undermining the Mediterranean diet approach [for example, “*Every day I eat fruit or freshly squeezed fruit juice,” “Regularly once a day would consume fresh and cooked vegetables,” “I eat pasta and rice almost every day (5 or more per week)”*]. The index ranged from 0 to 12, and consisted of a self-administered 16-question test. The validity of the KIDMED index is demonstrated by the evidence that a higher score is associated with the expected patterns of food and nutrient intake, representative of a good quality diet. The KIDMED index was inspired by tools developed previously for adults and the elderly. It is the first index that evaluates the adequacy of the Mediterranean food model in the population aged 2–24. The levels of physical activity were assessed using the Physical Activity Questionnaire for Older Children (PAQ-C). The questionnaire provides a general measure of physical activity for 8- to 20-year-olds. The PAQ-C is a self-administered questionnaire consisting of nine items rated on a five-point scale for about a 7-day (previous week) activity (e.g., “*In the last 7 days, during your physical education (PE) classes, how often were you very active (playing hard, running, jumping, throwing, etc.)?”*, “*In the last 7 days, on how many evenings did you do sports, dance, or play games in which you were very active?”*, and “*On the last weekend, how many times did you do sports, dance, or play games in which you were very active*?”). The average of the items is used to create the final PAQ summary score; a higher score indicates more active children or adolescents. Previous studies have supported the validity of the PAQ instrument for assessing general levels of physical activity. Validation studies have found the PAQ-C to be highly reliable (Cronbach alphas ranged from 0.72 to 0.88) (28).

In addition, questions were introduced on the type of housing (presence or absence of green spaces or terraces), on maintaining the relationships with peers *via* smartphone, in compliance with homeschooling, and on sleep quality.

### Statistical Analysis

The indicators belonging to the health-related quality of life were selected according to the analysis of the existing literature in the health and well-being of the adolescents (13–15).

Statistical data analyses were performed using SPSS (Version 22.0. Armonk, NY: IBM Corp). The data are presented as mean ± SD or as mean with 95% confidence interval (CI). Alpha was set at 0.05 and 2-sided *p*-values were reported. After CQ, variables related to changes in the health-related quality of life and lifestyle habits were analyzed using the Student's paired *t*-test, and the average differences were expressed as percentage of pre-quarantine mean values. A one-way within-group multivariate analysis of variance was performed to evaluate the overall changes in the scores of KIDSCREEN-52 domains and the lifestyle habits between SC and CQ. A mixed between–within ANOVA was conducted to evaluate the differences for changes after CQ, according to the gender, using sex as a control variable. Sensitivity analyses, stratified by gender, were run and the inter-group differences were evaluated by the Student's unpaired *t*-test.

## Results

### Socio-Demographic Characteristics of the Study Population

The descriptive data of the total cohort are depicted in Table 1. The results of socio-demographic parameters (e.g., housing location or housing typology) are the same both in SC and in CQ, as reported by the questionnaires in the AVATAR platform.

**Table 1:** Socio-demographic characteristic across the total cohort.

**Variables**	**Total cohort (*n* = 1,289)**
Age	12.53 ± 1.25
Male	622 (48.3%)
Female	667 (51.7%)
**HOUSING LOCATION**	
Rural	487 (37.8%)
Urban	802 (62.2%)
**HOUSING TYPOLOGY**	
Detached house	678 (52.6%)
Apartment	611 (47.4%)
**PRESENCE OF THE TERRACE**	
Yes	977 (75.8%)
No	312 (24.2%)
**PRESENCE OF THE GARDEN**	
Yes	984 (76.3%)
No	305 (23.7%)

*Data presented are mean value ± SD or number (%) of subjects*.

### Health-Related Quality of Life and Lifestyle Habits in SC and CQ Conditions

**Figure 1:**
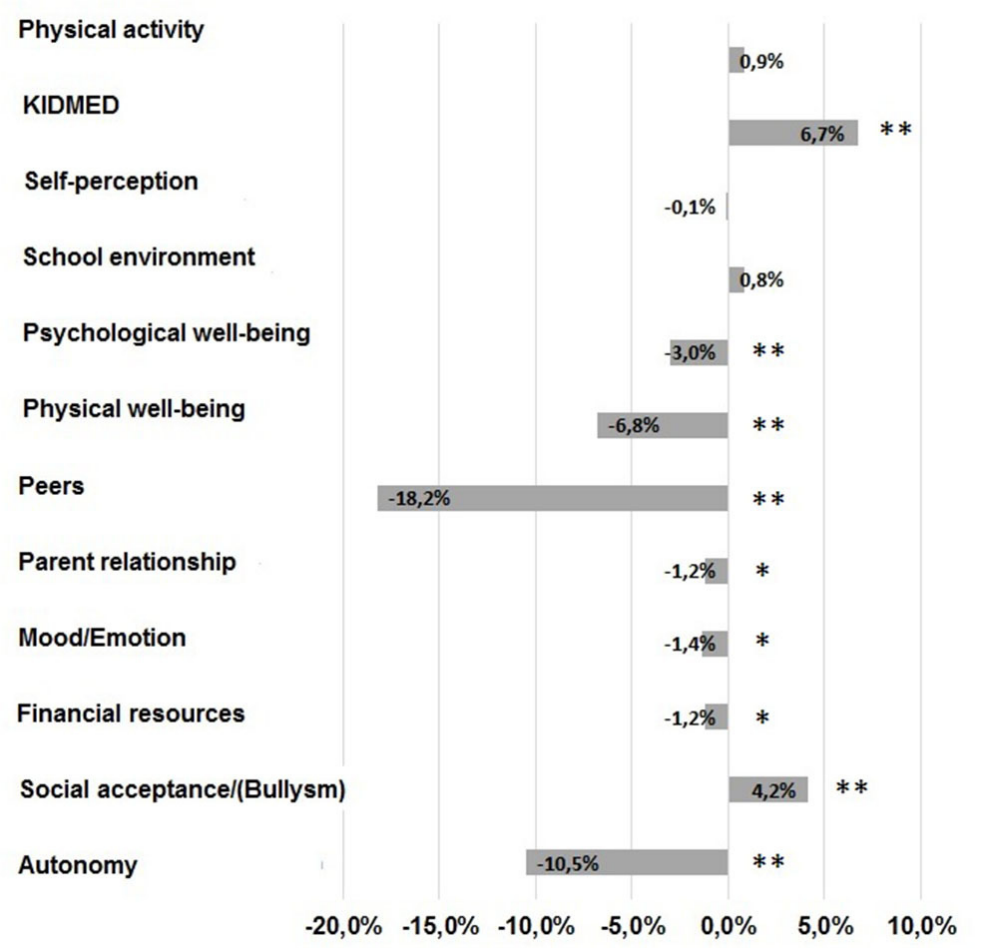
Change in the KIDSCREEN-52 domains and the lifestyle habits during COVID-19 quarantine with respect to the standard condition (SC) expressed as the percentage of pre-quarantine mean values (**p < 0.001, *p < 0.05).

Descriptive data on the health-related quality of life and lifestyle habits (diet and physical activity) in the study population, in SC and CQ are presented in Table 2, and the percentage changes are presented in Figure 1. Data from the KIDSCREEN-52 dimension are calculated as the mean T-scores according to the KIDSCREEN Group (25). There was a statistically significant change in the KIDSCREEN-52 domains and in the lifestyle habits during CQ compared to SC (Wilks' Lambda = 0.98, *F* = 3.6, *p* =0.01). In the CQ monitoring, the adolescents showed on average, a lower perception in the dimensions of the psychological well-being (mean change = −1.5, 95% CI: −2.0 to −1.0, *t*-statistics = −5.6, *p* < 0.001) and in the physical well-being (mean change = −3.2, CI: −3.6 to −2.8, *t* = −15.8, *p* < 0.001). The dimension of mood/emotion revealed that in the SC, the teenagers had higher mean scores than in the CQ (mean change = −0.7, CI: −1.2 to −0.2, *t* = −2.6, *p* = 0.008) as well as for autonomy (mean change = −4.9, CI: −5.5 to −4.4, *t* = −16.8, *p* = 0.001), understood as the opportunity to create his/her social and leisure time. In the CQ conditions, the perception of the financial resources compared to the initial situation is reduced (mean change = −0.6, CI: −1.1 to −0.1, *t* = −2.4, *p* = 0.018). In the social context assessment, adolescents, in the CQ compared to the SC, reported not only lower values both in the relationship with the family (mean change = −0.6, CI: −1.1 to −0.1, *t* = −2.3, *p* = 0.021) and with the peers (mean change = −9.2, CI: −10.0 to −8.5, *t* = −23.5, *p* = 0.001), but also exhibited a higher perception of social acceptance (mean change = 2.1, CI: 1.5–2.7, *t* = 7.2, *p* < 0.001). For lifestyle, during CQ, the adolescents developed higher adherence to the Mediterranean diet (mean change = 0.4, CI: 0.3–0.5, *t* = 6.4, *p* < 0.001) than during SC.

**Table 2:** KIDSCREEN-52 domains and lifestyle habits in the study sample of Standard Conditions (SC) and during COVID-19 quarantine (CQ).

**Variables**	**SC (*n* = 1,289)**	**CQ (*n* = 1,289)**	***p*-Value**
Physical well-being	46.9 ± 6.7	43.7 ± 6.9	<0.001
Psychological well-being	50.1 ± 9.5	48.6 ± 9.7	<0.001
Mood/emotion	48.4 ± 9.7	47.7 ± 9.5	<0.05
Self-perception	52.4 ± 10.6	52.4 ± 11	=0.80
Autonomy	47 ± 9.8	42 ± 8.5	<0.001
Parent relationship	51.1 ± 9.9	50.5 ± 10.3	<0.05
Financial resources	50.9 ± 9.4	50.2 ± 10.3	<0.05
Peers	50.6 ± 10.2	41.3 ± 12.7	<0.001
School environment	49.7 ± 8.8	50.1 ± 8.6	=0.07
Social acceptance (bullying)	50.2 ± 10.5	52.3 ± 9.6	<0.001
KIDMED	6.1 ± 2.6	6.5 ± 2.5	<0.001
PAQ-C	2.6 ± 0.7	2.7 ± 0.8	=0.30

*The data given as mean ± SD (95% CI). The data on the KIDSCREEN-52 dimension are calculated as the mean T-scores according to the KIDSCREEN Group. The p-values were calculated via Student's paired t-test. A one-way within-group multivariate analysis of variance was performed to evaluate the changes in scores between the SC and CQ including all the variables. There was a statistically significant difference on the combined variables in this analysis (Wilks' Lambda = 0.98, F = 3.6, p = 0.01)*.

### Gender Differences on Health-Related Quality of Life and Lifestyle Habits During COVID-19 Outbreak

Descriptive data of health-related quality of life and lifestyle habits in the total population divided by gender are presented in Table 3. In the female population, the CQ condition has highlighted a reduction in the perception of the physical (mean change = −2.5, CI: −3.1 to −2.0, *t* = −15.8, *p* < 0.001) and psychological well-being (mean change = −2.2, CI: −2.9 to −1.5, *t* = −6.0, *p* < 0.001), emotional background (mean change = −1.8, CI: −2.4 to −1.1, *t* = −5.3, *p* < 0.001), perception of self (mean change = −1.0, CI: −1.7 to −0.3, *t* = −2.9, *p* = 0.005), and autonomy (mean change = −4.9, CI: −5.7 to −4.1, *t* = −12.1, *p* < 0.001). In the social context, the score about the relationship with the peers (mean change = −8.8, CI: −9.8 to −7.9, *t* = −18.0, *p* < 0.001), and with family (mean change = −0.8, CI: −1.5 to −0.1, *t* = −2.1, *p* = 0.035) were lower in CQ than in SC, while the perception of social acceptance was higher (mean change = 1.7, CI: 0.9 to 2.4, *t* =4.4, *p* < 0.001). In the lifestyle assessment, in the CQ monitoring, adherence to the Mediterranean Diet (mean change = 0.5, CI: 0.3–0.6, *t* = 5.3, *p* < 0.001) and the physical activity level (mean change = 0.1, CI: 0.1–0.2, *t* = 4.5, *p* < 0.001) are increased. In the male cohort, a lowering perception of physical (mean change = −3.9, CI: −4.5 to −3.3, *t* = −12.9, *p* < 0.001) and psychological well-being (mean change = −0.8, CI: −1.5 to −0.1, *t* = −2.0, *p* = 0.049), and autonomy (mean change = −5.0, CI: −5.8 to −4.2, *t* = −11.7, *p* < 0.001) are reported in CQ with respect to SC. A better self-perception has been reported (mean change = 0.9, CI: 0.1–2.8, *t* = 2.1, *p* = 0.034). In the social setting, a decline in the relationship with the peer (mean change = −9.7, CI: −10.9 to −8.4, *t* = −12.9, *p* < 0.001) and a higher perception in the social acceptance (mean change = 2.6, CI: 1.7–3.4, *t* = 5.8, *p* < 0.001) are shown in the CQ condition. Lifestyle habits, during the lockdown, were characterized by improvement in adherence to the Mediterranean diet (mean change = 0.4, CI: 0.2–0.5, *t* = 3.8, *p* = 0.001) and reduction in the physical activity level (mean change = −0.1, CI: −0.2 to −0.1, *t* = −2.7, *p* = 0.008). Gender effect emerged for the dimension of the psychological well-being (F-statistic = 7.2, *p* = 0.007) and mood/emotion (*F* = 20.2, *p* = 0.001), in which females had on average, lower scores compared to males, while males showed a higher score in the dimension of the physical well-being (*F* = 11.9, *p* = 0.001). There was a gender difference with regard to self-perception (*F* = 12.2, *p* = 0.001), where females exhibited the lowest values than males. In the lifestyle assessment, males had lower scores in physical activity than females (*F* = 11.9, *p* = 0.001).

**Table 3:** Gender differences in KIDSCREEN-52 domains in standard conditions (SC) and during COVID-19 quarantine (CQ).

**Variables**	**Female SC (*n* = *x*)**	**Female CQ (*n* = *x*)**	***p*-value**	**Male SC (*n* = *x*)**	**Male CQ (*n* = *x*)**	***p*-value**	**Δ M vs. F *p*-value (Post-Pre)^#^**
Physical well-being	46.1 ± 6.7	43.6 ± 6.6	< 0.001	47.8 ± 6.5	43.9 ± 7.2	<0.001	<0.001
Psychological well-being	49.8 ± 10	47.6 ± 9.9	<0.001	50.3 ± 8.8	49.5 ± 9.4	<0.05	<0.01
Mood/emotion	48.1 ± 10	46.3 ± 9.8	<0.001	48.6 ± 9.3	49.2 ± 9	=0.19	<0.001
Self-perception	51.6 ± 10.9	50.6 ± 11.5	<0.01	53.3 ± 10.1	54.2 ± 10.2	<0.05	<0.001
Autonomy	46 ± 9.7	41.1 ± 8.1	<0.001	48.1 ± 9.8	43.1 ± 8.9	<0.001	=0.85
Parent relationship	51 ± 10.5	50.2 ± 10.5	<0.05	51.2 ± 9.3	50.8 ± 9.3	=0.26	=0.50
Financial resources	51.2 ± 9.4	50.6 ± 10.1	=0.09	50.5 ± 9.4	49.8 ± 10.5	=0.10	=0.88
Peers	50.4 ± 9.8	41.6 ± 11.3	<0.001	50.7 ± 10.5	41.0 ± 13.9	<0.001	=0.29
School environment	50.4 ± 8.8	50.9 ± 8.8	=0.08	49 ± 8.7	49.3 ± 8.4	=0.38	=0.67
Social acceptance (bullying)	50.6 ± 10.2	52.2 ± 9.7	<0.001	49.8 ± 10.7	52.4 ± 9.4	<0.001	=0.11
KIDMED	6.0 ± 2.6	6.4 ± 2.4	<0.001	6.2 ± 2.6	6.5 ± 2.6	<0.001	=0.43
PAQ-C	2.5 ± 0.7	2.6 ± 0.7	<0.001	2.8 ± 0.7	2.7 ± 0.8	<0.01	<0.001

*The data given as mean ± SD (95% CI). The data from the KIDSCREEN-52 dimension are calculated as the mean T-scores according to the KIDSCREEN Group*.

# *Δ [M (Post-Pre) vs. F (Post-Pre)] p-values were calculated via Student's unpaired t-test*.

### Role of Housing Conditions on the Health-Related Quality of Life During COVID-19 Outbreak

Concerning the influence of housing conditions on the dimensions of KIDSCREEN and lifestyle habits in lockdown condition, adolescents living in the village than those living in the city, had a greater reduction both in autonomy (mean ± SD: −10.8 ± 14.7 vs. −8.3 ± 13.6, *F* = 9.2, *p* = 0.002) and peer relationships (−6.1 ± 11.3 vs. −4.2 ± 10, *F* = 9.7, *p* = 0.002), in CQ than in SC. In addition, in the CQ monitoring, the perception of physical well-being was more reduced in those living in the city as compared to those living in the country (−3.6 ± 7 vs. −2.6 ± 7, *F* = 4.7, *p* = 0.03), in an apartment than in a detached house (−3.9 ± 7.4 vs. −2.6 ± 7.1, *F* = 9.5, *p* = 0.002), and in those who did not have green space (−4.4 ± 7.5 vs. −2.8 ± 7.2, *F* = 11.1, *p* = 0.001) than in the SC.

## Discussion

The present study explores for the first time, to our knowledge, the health-related quality of life in the early adolescents during COVID-19 outbreak, showing wide-ranging modifications, both from the psychological and social point of view, related to gender and housing characteristics. An important characteristic of this study, that makes it different from the previous ones, was the opportunity to assess the possible acute effects of quarantine with respect to a standard condition, since the same questionnaires have been filled out by the same students at the beginning of the school and during CQ (20–22, 29). Furthermore, this study conducted during the quarantine is different from the others assessing the long-term events. The main results of this study can be reassumed in the following points: (i) during the quarantine, early adolescents showed a lower perception in the psychological and physical well-being, with regard to dimensions related to mood/emotion, autonomy, and financial resources, mediated by housing and environmental conditions; (ii) in the social context, relationship with the family and with peers, as well as the perception of bullying were reduced in the CQ,; (iii) in the lifestyle assessment, during CQ, adolescents developed higher adherence to the Mediterranean diet; (iv) gender effect emerged for the psychological and physical well-being, mood/emotion, and self-perception.

Quarantine is one of the public health measures to avoid the spread of an infectious disease, but it also represents an extraordinary naturalistic experiment to study the psychophysiological effects of isolation, as well as on the quality of life and well-being. In human studies, the terms, quarantine and isolation are often used interchangeably, though quarantine is considered as the separation and restriction of the movement of people potentially exposed to a contagious disease, and isolation is the separation of people who have been diagnosed with a contagious disease from people who are not sick (30). Generally, quarantine determines the negative psychological effects, including emotional disturbances, depression, stress, low mood, irritability; but, as for animal models, these symptoms are described in studies assessing the impact longitudinally.

Filling the questionnaires during the quarantine allowed us to look at the acute response to a critical and unforeseen event. This is an important point since adolescents experience a transition period that is suddenly faced with a series of emotions, from fear to sadness and from thinking to impulsiveness. For this reason, adolescence is considered as a phase in which mental health problems could develop, but at the same time, it could be considered as an important resource for mitigating risks. This seemingly evolutionary disharmony, on the one hand, makes teenagers more likely and vulnerable to risk, but on the other hand, it allows them to adapt more easily to the environmental changes, thereby transforming fragility into a resilient behavior (31). Our results are in line with the dual nature of early adolescence, resilience, and vulnerability that coexist together. In fact, the acute modifications during the quarantine were in the psychological and social dynamics with a focus on the reduction in the perception of well-being, mood, capacity to create an individual identity, and awareness of financial resources. On the social level, home confinement induced a deficit in the relationship in different contexts, from peers to parents. Usually, family represents a principal setting with a protective role, associated with healthy behaviors, and more specially, in the quarantine condition, parents should be the closest and the best asset (32, 33). However, adolescents typically spend more time with the peers than with their family and this social relationship is pivotal to facilitating the transition to independent adults (15). Though quarantine could offer a good opportunity to improve the interaction between parents and adolescents, adolescents may, at the same time, feel more monitored and controlled, reducing the level of autonomy, crucial during adolescence. In addition, next to the lack of in-person contact with classmates, friends, and teachers, our results also showed a reduction in the perception of financial resources, creating a background that could have enduring effects, in line with our previous data (22). The data obtained from adolescents show that the perception of being in a low economical state, may promote depressive symptoms. This relation can be due to contextual risk factors, reduced social support, and risky health behavior (34).

Furthermore, the interaction between the housing quality and the perception of psychosocial well-being caused by home confinement could further aggravate the negative effects on the physical and mental health of adolescents. However, these changes, which apparently, in the acute phase, could describe an allostatic load condition, which could instead result in an adaptive response, from the perspective of the daily emotional experiences of adolescents (35). In other words, the changes shown by our results could be the acute effect of an increased stress reactivity that would lead to resilience in the chronic phase. In fact, compared to other ages of life, adolescence is the period characterized by the greatest resilience (36). Notwithstanding, resilience may be content- and context-specific, making adolescents resilient for one type of risk, but unable to overcome other types of risk (37). In line with this perspective, our results underlined that there are not only negative changes in the psychosocial dynamics, but also improvement in the lifestyle habits as observed by the increased adherence to the Mediterranean diet, and a reduction in the perception of bullying. This presumed inconsistency questions how a factor could be considered as an exposure to risk for some constructs, and as a resource and a promoter for healthy behaviors.

Another important question that could account for the health-related quality of life during COVID-19 outbreak on adolescents was that, early Italian adolescents were also affected by the closure of educational opportunities that deprived them of social engagement with their peers and educators. If homeschooling was an immediate guarantee from the didactic point of view, on an educational and psychological level, this was not enough to preserve the quality of life of adolescents. School, as a context for health, has a crucial role not only from an educational perspective, but also in offering an opportunity for students to interact with peers, teachers, encouraging activities during the spare time, healthy habits, promoting social and emotional development, and integrating health promotion into the school curriculum.

Evidence obtained from other contexts, such as summer holidays in which adolescents were out of school, prolonged school closure, and home confinement, might have negative effects on the health of children, such as reduced physical activity, irregular sleep patterns, and weight gain (38).

This is probably due to social deprivation on the adolescent brain and behavior. Early adolescence, in fact, can be considered as a sensitive period for social growth, dependent on the development of the social brain, but physical distancing measures radically reduces the opportunities to engage in face-to-face social contact outside their household, compromising not only peer interaction but also the vital aspect of development (10, 39).

Lastly, considering gender differences, we observed that the psychological well-being, mood, and self-perception decreased significantly more in females than in males. By looking into these results, interesting evidence shows that in males, when compared to females, the perception of self and mood increased, while the score in the dimension of physical well-being decreased in line with a reduction in the physical activity. This dichotomy between the psychological and physical constructs is in line with the previous studies, conducted not in the same confined situation, where adolescent girls exhibited higher levels of depressed mood and anxiety than boys (40, 41).

The main limitation of the study concerns the method of data acquisition, which for obvious experimental reasons, differed in two phases. In the SC, since the questionnaires were completed during a school class; the school classroom environment might have biased the responses of the students. During CQ, although questionnaires were completed during distance learning, in the presence of a teacher, they were conducted in the housing context. Moreover, the study group consisted of early adolescents and cannot be considered representative of all adolescents.

Furthermore, we do not know whether the consequences of social distancing were also associated with other experienced stressors or other variables, or perceived the effect of the isolation, by adolescents during the COVID-19 crisis, including, for example, the economic situation of the family.

In conclusion, the present study showed that the health-related quality of life during the quarantine assessment is changed in its psychosocial dimensions, from mood, self-esteem, and social relationships.

Special guidelines for early interventions should be issued to adolescents, parents, teachers, psychologists, and other stakeholders, with particular attention to school environment, considered as “context of socialization” that influences the developmental outcomes of students. The importance to assess the health-related quality of life during quarantine has the potential practical impact to adopt preventive strategies to avoid the occurrence of long-term health disturbances induced by the COVID-19 outbreak.

## Data Availability Statement

The raw data supporting the conclusions of this article will be made available by the authors, without undue reservation.

## Ethics Statement

The studies involving human participants were reviewed and approved by the Internal Ethics Committee of each participating School, in accordance with Italian law. Written informed consent to participate in this study was provided by the participants' legal guardian/next of kin.

## Author Contributions

FM: conceptualization, writing-original draft, methodology, and review and editing. PP: software, formal analysis, and review and editing. CD, GT, and AC: methodology, software, and data acquisition. MP and CV: conceptualization, and review and editing. AP: conceptualization, writing-original draft, methodology, review and editing, and supervision. All authors contributed to the article and approved the submitted version.

## Conflict of Interest

The authors declare that the research was conducted in the absence of any commercial or financial relationships that could be construed as a potential conflict of interest.
